# Supplementary material for: Apelin Promotes Prostate Cancer Metastasis by Downregulating TIMP2 via Increases in miR-106a-5p Expression
Source: Cells. 2022 Oct 19;11(20):3285. doi: 10.3390/cells11203285 (PMC9600532; doi:10.3390/cells11203285)
Supplement: Supplementary file 1 [file cells-11-03285-s001.zip › cells-1909423-supplementary.pdf]

Supplementary Files

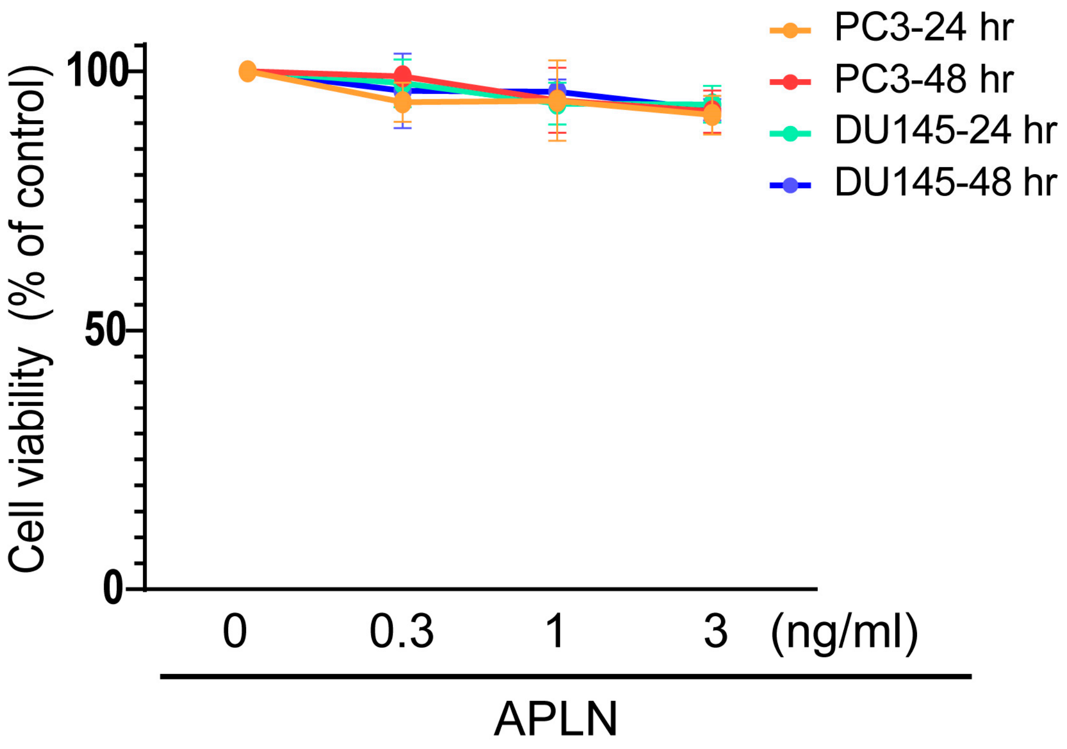

Supplementary Figure S1. Role of apelin in the cell viability of PC3 and DU145. PC3 and DU145 were incubated with different concentrations of apelin for 24 h and 48 h. Cell viability were examined by MTT assay (n=4).

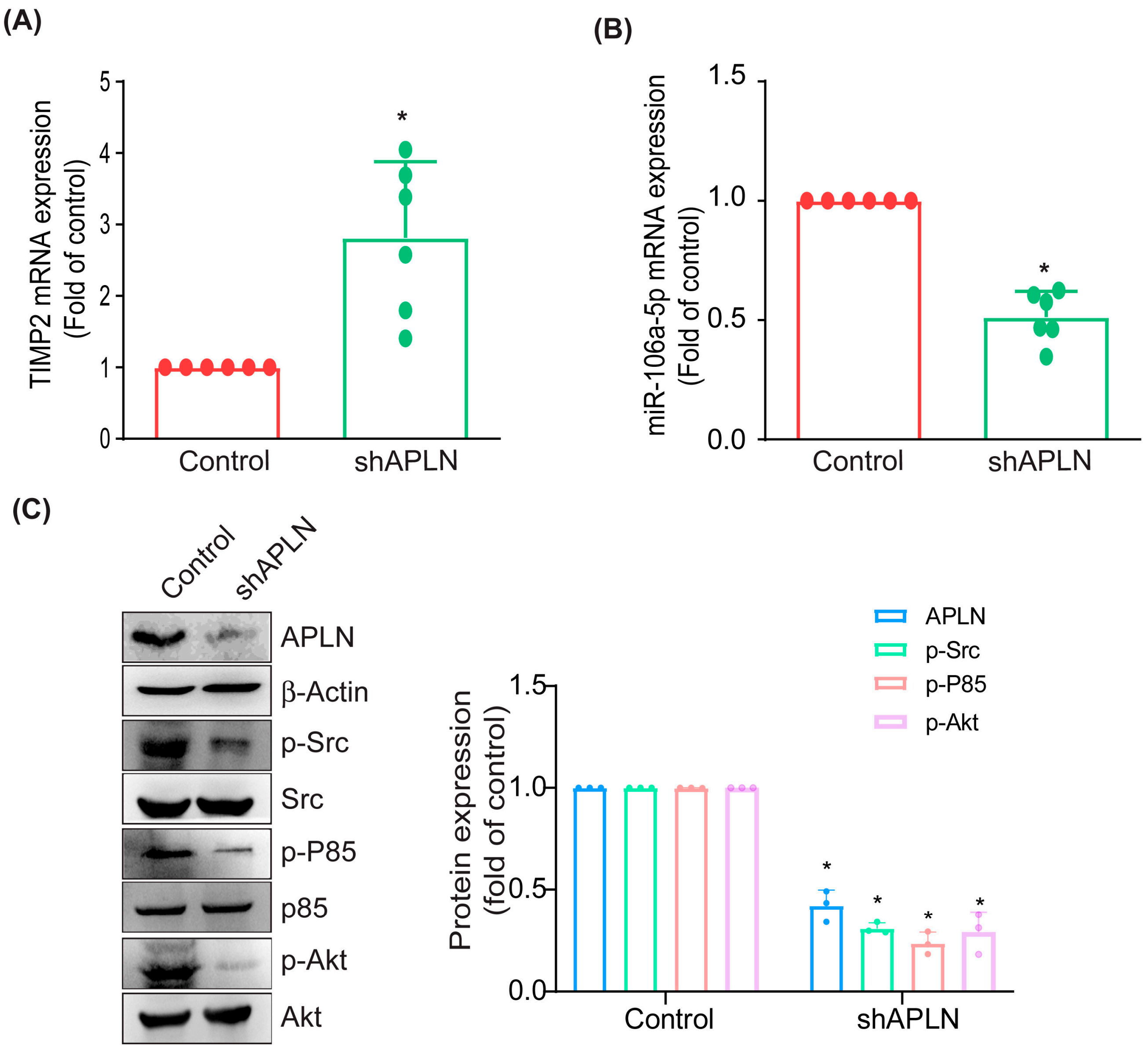

Supplementary Figure S2. Knockdown Apelin in PC3 cells down-regulated miR-106a-5p, p-Src, p-P85 and p-Akt expression, but up-regulated TIMP-2 expression. PC3 cells were transfected with apelin shRNA, then miR-106a-5p, p-Src, p-P85, p-Akt and TIMP-2 expression were examined by qPCR (n=6) and Western blot (n=3). * $P < 0.05$ compared with the control group.

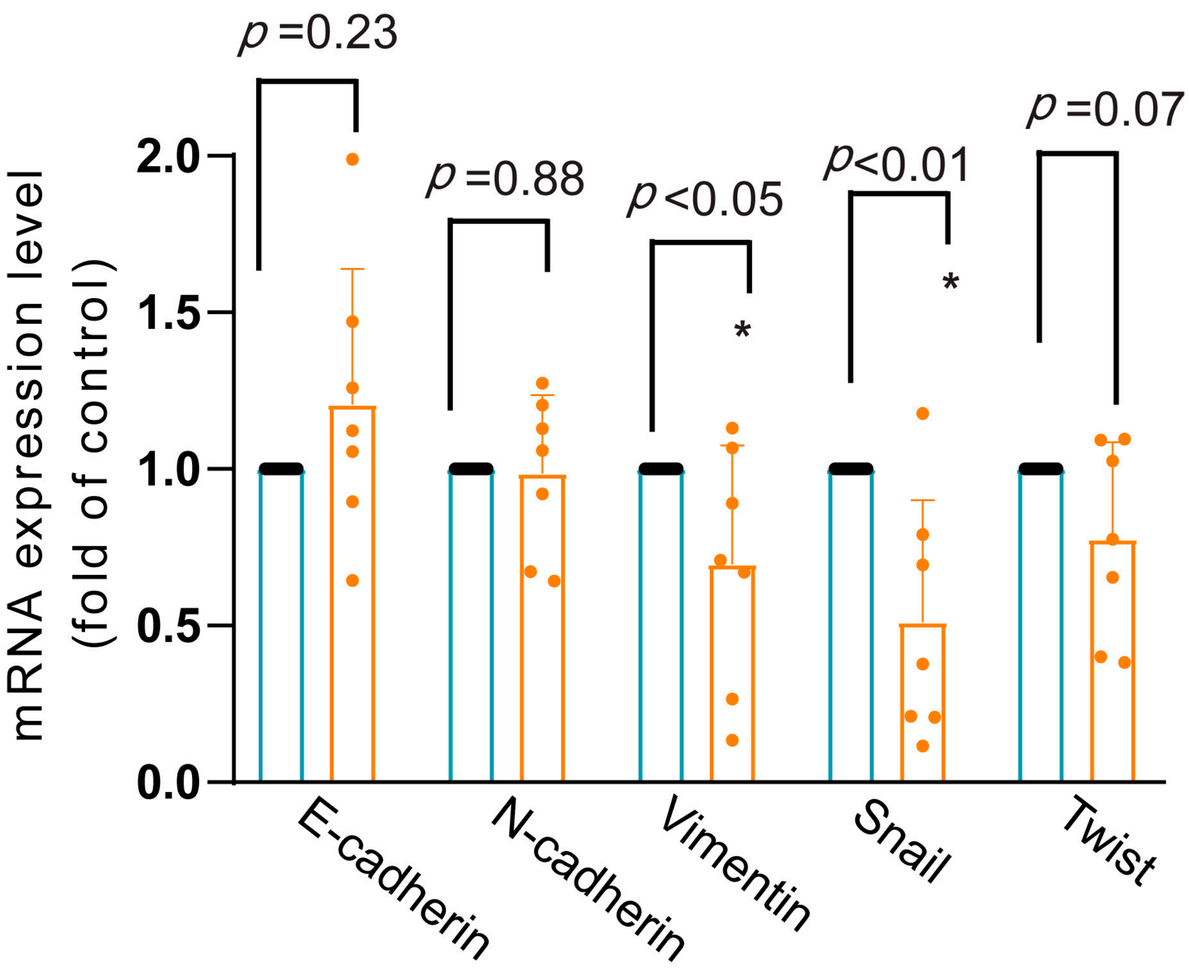

Supplementary Figure S3. Apelin did not significant effect the epithelial-mesenchymal transition of PC3 cells. Treatment with apelin (3 ng/ml) in PC3 cells for 24 h, the EMT markers expression was examined by qPCR (n=7). * *P* < 0.05 compared with the control group.

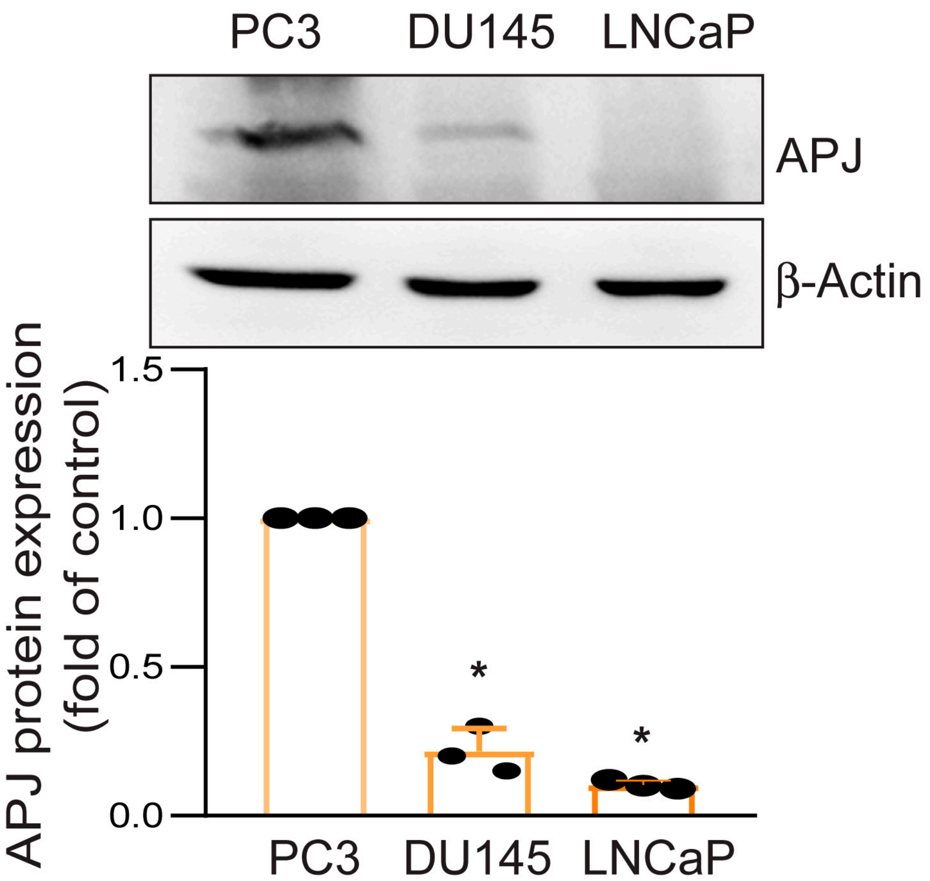

Supplementary Figure S4. The apelin receptor(APJ) levels in PC3, DU145, and LNCaP cells. The protein lysate of PC3, DU145, and LNCaP were collected and APJ expression were examined by Western blot assay (n=3). * $P < 0.05$ compared with the control group.

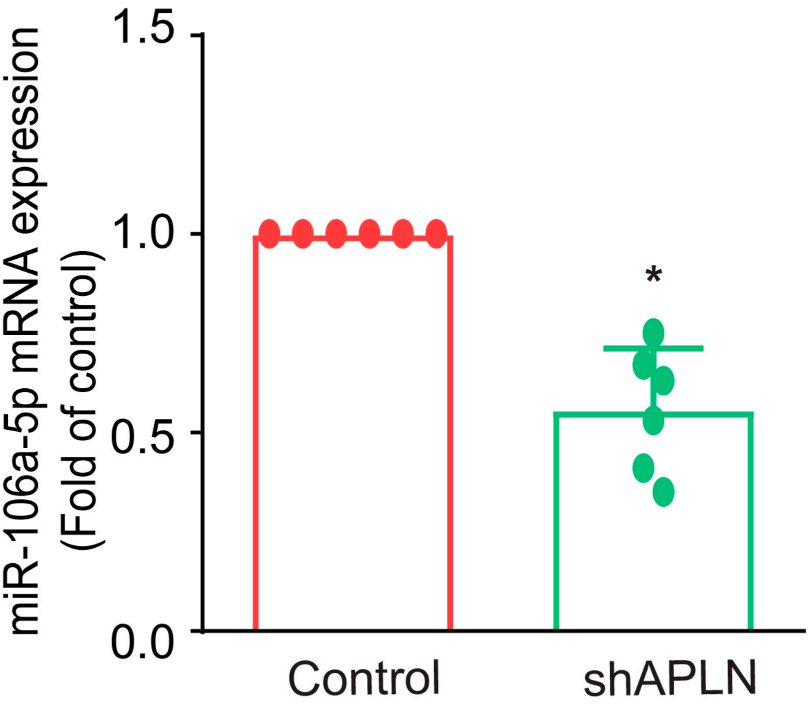

Supplementary Figure S5. miR-106a-5p was down-regulated in APLN knock-down tumor tissues. PC3-Luc or PC3/sh-APLN-Luc group tumors were collected and mRNA were extracted. The miR-106a-5p was determined by qPCR (n=6). * *P* values obtained by unpaired two-tailed t-test. * *P* < 0.05 compared with the control group.
